# Effects of heat exposure on self-reported heat strain symptoms among pregnant and postpartum women in South Africa

**DOI:** 10.1038/s44482-026-00036-4

**Published:** 2026-09-13

**Authors:** Clara Sofia Heil, Shobna Sawry, Günter Alce, Jean le Roux, Pascalia Munyewende, Celeste Madondo, Rose Refilwe Lamola, Sibusiso Mkwananzi, Ijeoma Solarin, Perkins Watambwa, Koen van der Sanden, Isabelle L. Lange, Susanne Frennert, Boris Kingma, Veronique Filippi, Stanley Luchters, Chuansi Gao, the HIGH Horizons Study Group, Günter Alce, Perkins Watambwa, Boris Kingma, Veronique Filippi, Chuansi Gao, Stanley Luchters, Matthew Chersich, Gloria Maimela, Celeste Madondo, Shobna Sawry, Mags Beksinska, Lebohang Radebe, Darshnika Lakhoo, Ijeoma Solarin, Craig Parker, Pascalia Munyewende, Jean Le Roux, Rose Lamola, Sibusiso Mkwananzi, Nicholas Brink, Jakob Eggeling, Clara Heil, Olof Stephansson, Claudia Hanson, Nathalie Roos, Jeroen de Bont, Veronika Tirado, Mian Bao, Massimo Stafoggia, Federica Nobile, Anayda Portela, Jørn Toftum, Ilona Otto, Chloe Brimicombe, Katharine Wieser, Tobias Monthaler, Zeenat Sulaiman, Sohail Baloch, Aquinius Mung’atia, Fortunate Machingura, Jetina Tsvaki, Thabani Moronzie, Concilia Mutasa, Brian Mqondisi Sibanda, Tariro Chinozvina, Elizabeth Dangaiso, Jaspar Maguma, Bongani Mutimutema, Progress Chiworeka, Leslie Nyoni, Llyod Pisa, Jeffrey Dirawo, Tapiwa Nyakabau, Debra Jackson, Giulia Greco, Nasser Fardousi, Olimpia Lamberti, Isabelle Lange, Giorgia Gon, Jo Borghi, Paul Lokubal, Cherie Part, Christos Hadjichristodoulou, Barbara Mouchtouri, Maria Kyritsi, Michalis Koureas, Fani Kalala, Chara Bogogiannidou, Ioanna Voulgaridi, Ioannis Messinis, Christina Messini, Xara Skentou, Matthaios Speletas, Andreas Flouris, Vana Papaevagelou, George Daskalakis, Androniki Naska, Vassiliki Benetou, Anastasia Tania Konstantinidou, Koen van der Sanden, Birgit Kerstens

**Affiliations:** 1 Aerosol and Climate Laboratory, Division of Ergonomics and Aerosol Technology, Department of Design Sciences, Faculty of Engineering, Lund University, Lund, Sweden; 2 Wits RHI, Faculty of Health Sciences, University of the Witwatersrand, Johannesburg, South Africa; 3 Wits Planetary Health Research Division, Faculty of Health Sciences, University of the Witwatersrand, Johannesburg, South Africa; 4 Centre for Sexual Health and HIV/AIDS Research (CeSHHAR), Harare, Zimbabwe; 5 Netherlands Organisation for Applied Scientific Research (TNO), Soesterberg, The Netherlands; 6 London School of Hygiene and Tropical Medicine, London, UK; 7 Department of Neurology, TUM University Hospital and Center for Global Health, School of Medicine and Health, Technical University of Munich, Munich, Germany; 8 Department of Public Health and Primary Care, Ghent University, Ghent, Belgium; 9 Liverpool School of Tropical Medicine (LSTM), Liverpool, UK; 10 Wits Planetary Health, Faculty of Health Sciences, University of Witwatersrand, Johannesburg, South Africa; 11 Karolinska Institutet, Stockholm, Sweden; 12 Department of Epidemiology, ASL Roma, Roma, Italy; 13 World Health Organization, Geneva, Switzerland; 14 Technical University of Denmark, Lyngby, Denmark; 15 Wegener Center for Climate and Global Change, University of Graz, Graz, Austria; 16 Aga Khan Health Services Kenya, Nairobi, Kenya; 17 Laboratory of Hygiene and Epidemiology, Medical Faculty, University of Thessaly, Larissa, Greece; 18 Ghent University, Ghent, Belgium

**Keywords:** Diseases, Health care, Medical research, Physiology, Risk factors, Signs and symptoms

## Abstract

Heat exposure can harm pregnant and postpartum women, yet heat strain symptoms in this population remain underexplored. This study implemented a novel mobile application and assessed the environmental context of five self-reported heat strain symptoms: skin rashes, sleep disturbances, dizziness, thirst, and exhaustion among pregnant and postpartum women in Tshwane, South Africa. Symptoms were assessed through interviewer-administered questionnaires at three time points over six months (*N* = 201). Self-reported heat strain was analysed against maximum Wet Bulb Globe Temperature (WBGTmax) and air temperature (Tmax). An additional symptom, wetness sensation, was collected continuously via self-reporting using the application and related to the Universal Thermal Climate Index (UTCI). Generalised Estimating Equation models and General Linear Mixed Models accounted for repeated measures. Four of five symptoms showed significant associations with both WBGTmax and Tmax. Wetness sensation was significantly correlated with UTCI. Thresholds for heat strain symptoms identified through Receiver Operating Characteristic curves ranged between 18.53 °C and 26.16 °C WBGTmax, reflecting differences in perceived severity. Urine specific gravity showed inconsistent association with heat, likely due to measurement timing limitations. Although risk varied across symptoms and threshold modelling was exploratory, this study highlights the adverse health consequences of heat exposure for pregnant and postpartum women, which may inform future interventions.

## Introduction

As our world heats up, exposure to ambient heat extremes has become an increasingly critical public health concern, affecting large parts of the world population^1^. Climate projections suggest that in a business-as-usual climate change scenario, one third of the global population will experience a mean annual temperature (MAT) of >29 °C within the next fifty years^1^. Currently, such conditions are limited to 0.8% of the Earth’s land surface, primarily in the Saharan region^1^. Regions most vulnerable to the impact of heat are among the poorest in the world^2^, with South Africa facing increasing challenges, particularly those rooted in household demographics and inadequate infrastructure^3,4^. Among those most vulnerable to extreme heat exposure are pregnant women^5^. Heat stress is caused by six factors, four of which are considered environmental parameters, including air temperature, humidity, air velocity and radiant temperature, and two personal parameters, including metabolic rate and clothing. Heat strain represents the body’s physiological responses to heat stress and heat illness describes adverse health outcomes when thermoregulation cannot compensate for the heat load, typically occurring with high heat strain^6,7^. Heat strain during pregnancy is often associated with headaches, dizziness, nausea, dry mouth and fatigue^8–10^. Other research also reports sleep difficulties, skin irritations, and difficulties performing basic daily activities and/or maintaining self-care^11^. Additionally, a well-documented link between extreme heat and adverse pregnancy and birth outcomes has been established, with findings showing heightened risk of preterm birth, stillbirth, low birth weight, gestational diabetes mellitus, hypertensive disorders, amongst others^12,13^. Preliminary research has demonstrated that extreme heat can trigger harmful physiological responses during pregnancy^14^. Once the core body temperature reaches an individual’s thermal load, adverse health outcomes, including heat illness, heat stroke and heat syncope, become increasingly likely^9^.

Pregnancy presents additional challenges to thermal regulation, as the placenta and foetus impose increased physiological demands on the maternal body. An elevated basal metabolic rate during pregnancy increases heat production, leading to intensified physiological responses and heightened thermal sensations^15^. An expert meeting found that during pregnancy, protective adaptive physiological measures in pregnant women include lowered sweating threshold, increased vasodilation and increased plasma volume, which collectively aim to improve heat dissipation^14^. However, during extreme heat exposure, these protective mechanisms may lose efficiency, leading to a higher risk of heat strain^16^, with previous qualitative research reporting that pregnant women feel the heat more acutely and describe greater difficulties to cool their bodies during hot weather^8^. When the maternal core temperature rises, it can lead to adverse outcomes for both the pregnant woman and the foetus. Foetal core temperature typically measures 0.5 °C higher than that of the maternal core temperature, making the foetus directly dependent on the mother’s ability to regulate heat^14^. Furthermore, the metabolic rate increases throughout pregnancy and rises to 120% in the third trimester. Changes in thermal sensation are therefore most pronounced further along the pregnancy, with individuals reporting higher thermal sensation in the third trimester, compared to earlier pregnancy stages. In low- and middle-income countries (LMICs), women frequently remain physically active into late stages of pregnancy due to their responsibilities surrounding motherhood (childcare, cleaning, cooking), and domestic, income-generating work^8,11^.

Heat-related health adversities have also been reported among postpartum women. Although physiologically distinct from pregnant women, postpartum women also differ from the general population. The early postpartum period is characterised by substantial hormonal shifts^17^, altered sleep patterns^18^ and lactation, which increases metabolic demands^19^. All of these parameters may influence thermoregulation, hydration states and thermal perception, ultimately changing the vulnerability to heat. Recent research further suggests that high ambient temperatures may negatively affect breastfeeding practices and maternal mental health and wellbeing^20,21^. In some settings, postpartum women also face increased domestic or caregiving obligations, including time spent obtaining water, food or firewood, which may further increase heat exposure^21^. Despite these vulnerabilities, postpartum women remain underrepresented in heat-health research. Given the important physiological differences between pregnancy and the postpartum period, we analysed these groups separately throughout the study, and assessed whether a significant difference between the groups could be found. As many pregnant and postpartum women face prolonged and unavoidable heat exposure^1^, targeted adaptation strategies must be developed and implemented to support their health and wellbeing, particularly among historically marginalised communities in urban and peri-urban settings experiencing structural inequality with inadequate access to protective infrastructure^22^. Behavioural adaptations to heat include actions such as seeking shade, reducing physical activity, removing layers of clothing and using water to cool down. These responses can also be supported through timely information and alerts through digital health tools that can be delivered through scalable, real-time digital and mobile platforms in LMIC settings.

Currently, many thresholds at the intersection of heat and health are derived from epidemiological analyses of historical temperature records and associated adverse health outcomes^23^. These thresholds signal the temperature levels at which mortality or morbidity risk increases, enabling alerts to be issued before the critical temperatures are reached^23^. Symptoms such as dizziness and exhaustion often precede more serious outcomes^24^ and can therefore serve as early warning indicators. Using these heat strain symptoms as thresholds for intervention may help prevent clinical complications and protect maternal wellbeing and daily functioning. There are, however, only a few studies that have identified thresholds based on heat strain, rather than clinical health consequences^25^. We aim to examine the relationship between heat exposure and heat strain symptoms in pregnant and postpartum women, and to identify exposure thresholds that may inform early warning systems (EWS) and digital health interventions. This study thereby provides empirical evidence on heat-related risks among pregnant and postpartum women and characterises how these risks change with increasing heat exposure. We hypothesise that higher heat exposure (WBGT) is associated with increased odds of self-reported heat strain symptoms among pregnant and postpartum women, with identifiable thresholds at which risk increases.

## Results

Of the 201 participants who enroled, there were seven early discontinuations: five could not be contacted after the enrolment visit, one was unwilling to install the app, and one was not interested in continuing beyond enrolment with no reason provided. None of the seven withdrew consent for their enrolment data to be used. Data from 201 participants were included in the analyses; 54 participants did not complete all follow-up visits. Participants who dropped out did not differ significantly at baseline in terms of age (independent samples t-test) or general health, chronic health conditions, perceived indoor heat, housing type (formal vs informal dwellings) or whether they perceived heat to be a serious issue for themselves or their infants (chi-square tests), with all *p*-values ≥ 0.05 based on chi-square tests for categorical variables. There were some indications of differences by education, with a non-significant overall association (chi-square test, *p* = 0.088) but evidence of a linear trend (*p* = 0.017), suggesting lower dropout among more highly educated participants.

### Descriptives

Measured at baseline, the average participant across cohorts was 159.8 cm tall, pregnant women weighed 75.8 kg on average (*SD* = 15.0), and postpartum women weighed 72.8 kg (*SD* = 16.6). Pregnant participants were on average 29.4 years of age at baseline; postpartum participants were 28.7 years of age. Not being employed but looking for work was the most commonly reported employment situation; 41% of pregnant participants and 57% of postpartum participants (Table S1). General health at baseline was rated as good by most participants (55% pregnant, 61% postpartum) (Table S2). For the pregnant cohort, participants’ gestational age at enrolment ranged from 13 to 26 weeks (*median* = 20, *IQR* = 15-23). For the postpartum cohort, the baby’s birth weights ranged between 750 g and 4500 g (*median* = 3095 g, IQR = 2700 g to 3445 g). For more details on maternal demographics and infant data, see Table 1. Most participants noted to be more tired than usual on hot days (pregnant 78%, postpartum 70%) and more irritable or moody (40% and 43% respectively; Table 2). For information on pregnancy history and characteristics, see Tables S3 and S4. Time spent outdoors between 6 am and 6 pm ranged from 0 to 11 hours, with a median of 2 hours for pregnant women and 4 hours for postpartum women (Table 3). Most participants lived in a house or brick/concrete structure on a separate strand (pregnant 57%, postpartum 64%) or an informal dwelling (pregnant 34%, postpartum 25.8%) (Table 4). Around half of the participants reported it being hotter indoors compared to outdoors (Table S5). For the 14-day average maximum WBGT, we found a temperature range of 16.0 °C to 28.8 °C, and for the 14-day averaged maximum temperature, this range was 18.0 °C to 33.2 °C. Descriptive statistics on both heat-related symptoms gathered through the questionnaires and in-app questions can be found in Tables S6 and S7.

**Table 1:** Maternal demographics and infant data

Maternal demographics and infant data at enrolment						
		*N*	Min	Max	Mean	SD
**Maternal height**	Pregnant	100	148	183	161	7
	Postpartum	101	146	173	159	6
**Maternal weight**	Pregnant	100	49	119	76	15
	Postpartum	101	45	133.5	73	17
**Maternal age**	Pregnant	100	20	43	29	6
	Postpartum	101	18	45	29	6
**Gestation**	Pregnant	91	13	26	19	4
**Infant birth weight**	Postpartum	100	750	4500	3084	585

Maternal height in cm, weight in kg, gestation in weeks at recruitment, birth weight of infant in grams at recruitment, self-reported.

Missing information on gestation from nine participants, Missing information on infant birth weight from one participant.

**Table 2:** Self-reported heat-related symptoms reported by pregnant women (*N* = 100) and postpartum women (*N* = 101)

On a typical hot day, how do you feel			
		*n*/*N*	Percent
No different than usual*	Pregnant	6/100	6.0
	Postpartum	12/101	11.9
**More tired than usual**	Pregnant	78/100	78.0
	Postpartum	71/101	70.2
**Irritable or moody**	Pregnant	40/100	40.0
	Postpartum	43/101	42.6
**Unable to concentrate**	Pregnant	38/100	38.0
	Postpartum	19/101	18.1

*No different than usual functioned as a mutually exclusive (“none of the above”) option, which automatically prevented participants to select more than this response option. Other response options could be selected simultaneously.

Please note that these numbers do not add to the total number of participants, since multiple options could be selected. The total number of participants *N* = 201.

**Table 3:** Daily time spent outdoors and on heavy work, both measured in hours

		*N*	Min	Max	Median	IQR
**Time spent outdoors (hours)**	Pregnant	100	0	11	2	1-4
	postpartum	101	0	11	4	2-6
**Time spent on heavy work (hours)**	Pregnant	100	0	8	1	0.5-2
	postpartum	101	0	6	2	1-3

This data was collected at baseline.

**Table 4:** Type of housing alternatives selected by participants

Type of housing			
		n/N	Percent
House or brick/concrete structure on a separate strand	Pregnant	57/100	57.0
	Postpartum	64/101	63.4
Flat or apartment in block of flats	Pregnant	2/100	2.0
	Postpartum	0/101	0.0
House, flat or room separate from main dwelling in backyard	Pregnant	5/100	5.0
	Postpartum	6/101	5.9
Informal dwelling or shack in backyard	Pregnant	9/100	9.0
	Postpartum	14/101	13.9
Informal dwelling not in backyard*	Pregnant	25/100	25.0
	Postpartum	12/101	11.9
Room or flat which is part of main dwelling property	Pregnant	1/100	1.0
	Postpartum	3/101	3.0
Other	Pregnant	1/100	1.0
	Postpartum	2/101	2.0

This data was collected at baseline.

### Heat strain symptoms collected through questionnaire related to WBGTmax

**Fig. 1:**
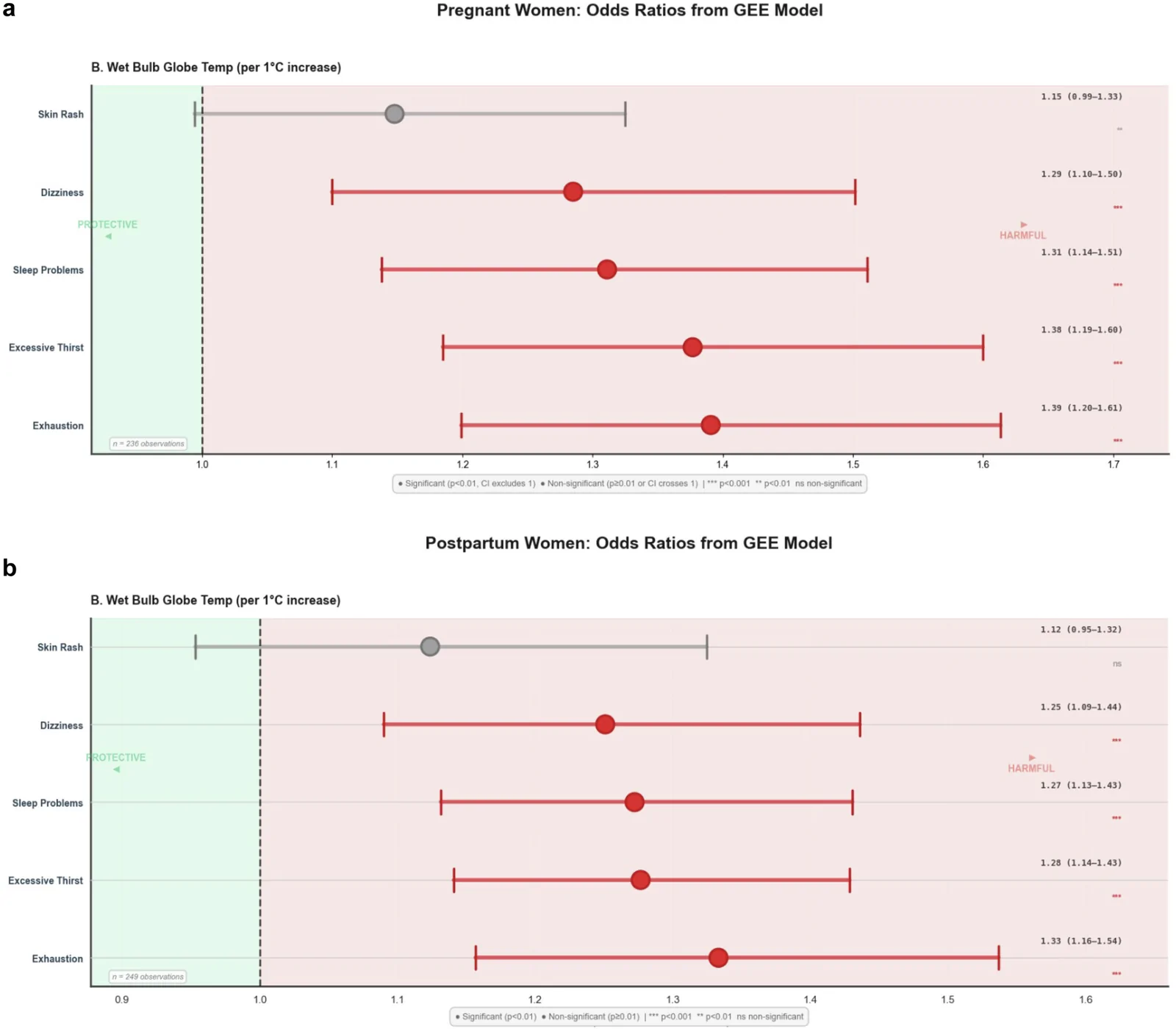
Association between WBGTmax and heat strain symptoms among pregnant and postpartum women. **a** Forest plot showing Odds Ratio (OR) and confidence intervals for the adjusted models for the pregnant cohort using Wet Bulb Globe Temperature (WBGTmax) as a heat indicator. **b** Forest plot showing Odds Ratio (OR) and confidence intervals for the adjusted models for the postpartum cohort using Wet Bulb Globe Temperature (WBGTmax) as a heat indicator. For both cohorts, Bonferroni correction was applied, accounting for multiple testing. Reference: no symptom (0), symptom present (1). Logit link, exchangeable correlation.

After correcting for multiple testing, all heat symptoms, except for skin rash, were significantly associated with WBGTmax across cohorts, both in the unadjusted and covariate-adjusted model (Fig. 1a and 1b for the adjusted WBGTmax model and Table S11 for the unadjusted WBGTmax model). In the Tmax model, again all heat symptoms, except for skin rash in the postpartum cohort, were significant in both the adjusted and unadjusted models, also considering the Bonferroni correction (Tables S11 and S12 and Figures S1a and S1b). In the adjusted models, six covariates were added, including maternal age, parity, general health (self-rated), type of home, time spent on heavy work per day, and whether it felt hotter indoors or outdoors. The covariates were tested for collinearity; with multicollinearity diagnostics indicated no concern for collinearity between variables regardless of cohort (VIF < 2.5 for all covariates), see Table S8. At the overall group level in the pregnant cohort, no covariates were significantly associated with WBGTmax for skin rash, sleep disturbances or thirst. For dizziness, however, hotter indoor conditions modified the WBGTmax association and housing type influenced exhaustion (Table 4 for more details on housing type). In the postpartum cohort, again no covariates were significant for sleep disturbance or thirst, while the hotter indoor condition significantly affected the relationships with skin rash, dizziness and exhaustion symptoms. Dizziness was additionally influenced by the amount of heavy work performed. Exhaustion was further associated with housing type and parity. For detailed results, see Table S9. Finally, an interaction analysis showed that dizziness was the only heat strain symptom that differed significantly between the pregnant and postpartum cohorts (*Wald χ²* *=* 4.664, *p* = 0.031), more details in Table S10. All associations between heat exposure and heat strain symptoms were significant in the pooled interaction analysis (Table S13).

**Fig. 2:**
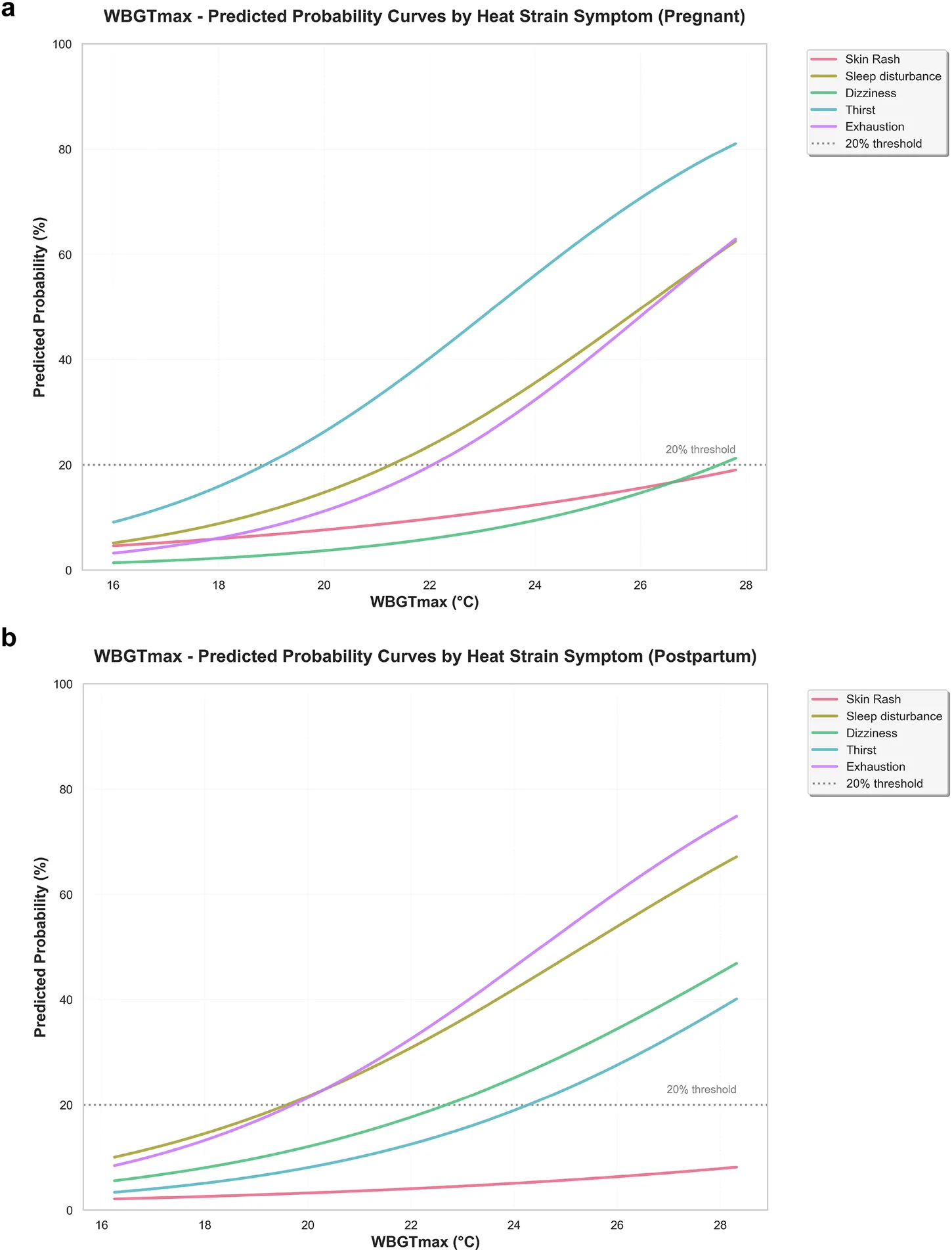
Predicted probability of of heat-related symptoms by WBGTmax among pregnant and postpartum women. **a** Predicted probability of five heat-related symptoms; skin rash, sleep disturbance, dizziness, thirst and exhaustion by maximum Wet Bulb Globe Temperature (WBGTmax) for pregnant cohort. Figure S3a highlights thresholds derived directly from this predicted probability curve. **b** Probability of five heat-related symptoms; skin rash, sleep disturbance, dizziness, thirst and exhaustion by maximum Wet Bulb Globe Temperature (WBGTmax) for postpartum cohort. Figure S3b highlights thresholds derived directly from this predicted probability curve.

We further calculated the odds of an increase in the probability of perceiving a symptom for every 1 °C WBGT increase, and converted log odds to odds ratio (OR). Across the observed range of 16.0 to 28.8 °C, on average, for pregnant women the odds of reporting heat rash increased by 15% for every 1 °C increase in max WBGT. Sleeping disturbance increased by 31%, dizziness by 29%, thirst by 38% and exhaustion by 39%. For postpartum women, odds of reporting heat rash increased by 12%, sleep disturbance by 27%, dizziness by 25%, thirst by 28% and exhaustion by 33% (for more details see Figs. 1a, 1b, 2a, 2b). Finally, the ROC analysis determined data-driven thresholds, which varied between pregnant and postpartum participants, and across symptoms. All ROC analyses were significant, except for skin rash in the pregnant cohort. For pregnant women, thresholds ranged from 21.19°C WBGT for thirst and exhaustion to 25.07 °C WBGT for dizziness, while for postpartum women thresholds ranged from 18.53°C WBGT for skin rash to 26.16 °C WBGT for dizziness (Table 5). For Tmax thresholds, see Table S14. Furthermore, dehydration, measured using urine specific gravity (USG), was not significantly associated with WBGTmax *ρ* = 0.063, *p* = 0.149, *N* = 528, but it was significantly associated with Tmax *ρ* = 0.187, *p* < 0.001, *N* = 528. The same trend could be identified for stratified analyses (Table S15).

**Table 5:** Receiver operating characteristics (ROC) analysis identifying WBGT thresholds for heat strain symptoms among pregnant and postpartum participants

Heat strain symptom	Cohort	AUC	95% CI	Optimal WBGT cutoff
**Skin rash**	Pregnant	0.522	0.437–0.606	24.40
	Postpartum	0.610	0.518–0.702	18.53
**Sleep disturbance**	Pregnant	0.701	0.637–0.765	23.00
	Postpartum	0.666	0.601–0.731	25.07
**Dizziness**	Pregnant	0.645	0.579–0.710	25.07
	Postpartum	0.650	0.538–0.717	26.16
**Thirst**	Pregnant	0.728	0.658–0.798	21.19
	Postpartum	0.689	0.619–0.759	25.07
**Exhaustion**	Pregnant	0.732	0.667–0.798	21.19
	Postpartum	0.707	0.641–0.773	23.45

The null hypothesis for ROC analysis is AUC = 0.5, indicating no discriminatory ability. *AUC* area under the curve

### Wetness sensation collected through an in-app survey related to UTCI

For the data collected through the EWS, the multinomial GLMM was conducted based on 6488 observations, with participant ID as a random effect accounting for repeated measures and showed that temperature significantly affected sweating intensity (*p* < 0.001). The frequency of responses varied from 1 to 440, with a median response of 67 and an IQR of 112 (Fig. S2 for more details). Compared to the lowest sweating category (dry) used as reference, higher UTCI temperatures increased the odds of reporting moderate (OR 1.09, 95% CI 1.06–1.12), strong (OR 1.08, 95% CI 1.05–1.12), or extreme sweating (OR 1.11, 95% CI 1.05–1.17), with estimates expressed per 1 °C increase. The strongest association was seen for the highest sweating category (Table 6). Furthermore, random effects were significant, highlighting great between-participant heterogeneity, particularly for the highest sweating category (see Table S16).

**Table 6:** OR distribution, multinomial generalised linear mixed model (GLMM) using UTCI and four different categories of sweating: dry, a little wet, wet and extremely wet to assess level of wetness on skin

Sweat Rating	*n*	OR	Var(Intercept)	95% CI lower	95% CI upper
Alittle wet	1111	1.09	1.31	1.06	1.12
Wet	1213	1.08	1.64	1.05	1.12
Extremely wet	299	1.11	3.06	1.05	1.17

Reference category: dry, *N* = 3865.

## Discussion

Our study demonstrates that both 14-day average outdoor maximum Wet Bulb Globe Temperature (WBGTmax) and air temperature (Tmax) are consistently associated with increased risk of heat strain symptoms in pregnant and postpartum women. These associations remained significant after adjusting for covariates and pooled analyses. Skin rash is the only heat strain symptom that, after correcting for multiple testing, was not significantly associated with heat. Across the observed WBGT range (16 °C to 29 °C), each 1 °C increase in WBGT, on average, was associated with a 15% to 39% increase in odds of experiencing a heat symptom for pregnant women and a 12% to 33% increase for postpartum women. Although these estimates are relatively large, they appear plausible and broadly consistent with previous literature. A recent study in the general population reported a 41% increase in odds of heat illness per 1 °C increase in WBGT^26^. Another study examining foetal heat strain found a 20% increase in odds of maternal heat strain on foetal heat strain per 1°C UTCI^9^. Finally, a recent systematic review and meta-analysis reported that adverse maternal health outcomes, such as gestational diabetes mellitus, increased by 28% with higher heat exposure^13^. Nonetheless, it is important to remember that our findings build on self-reported heat strain symptoms and therefore likely are not an accurate depiction of underlying ongoing biological mechanisms. Furthermore, the reported ORs should be interpreted as relative measures of association rather than direct estimates of absolute risk increase. The practical public health relevance of these estimates also depends on baseline frequency of each symptom outcome. Therefore, the reported effect estimates should be interpreted with caution. Using UTCI, we further found that heat was strongly associated with sweating sensation, with an 8-11% increase in the odds of reporting wet rather than dry skin per 1 °C UTCI. Nevertheless, although sweating indicates that heat stress exceeds thermoneutral levels, it primarily reflects a normal thermoregulatory response rather than necessarily indicating heat strain. Furthermore, in this population, which is likely acclimatised to the local environment, increased sweating may partly represent an adaptive response. Heat measured using WBGTmax was not linked to dehydration (USG), whereas heat measured using Tmax was. According to recent literature, WBGT is a suitable predictor of hydration status and heat perception^27^. The non-significant finding is likely due to the timing of urine sampling during routine health care visits, rather than collecting samples early in the morning or after physical exertion (e.g., a workday), where dehydration is typically measured and is more detectable^28^. Therefore, the null finding for dehydration may reflect the measurement timing rather than a true absence of effect.

We identified the optimal heat thresholds through the ROC analysis. All heat thresholds for pregnant women were lower compared to postpartum women, except for the skin rash thresholds. The lower thresholds make sense, as during pregnancy, women are particularly vulnerable to complications due to heat exposure. Experiencing these heat strain symptoms at lower heat levels would further support this interpretation. Overall, some heat symptoms appear less suitable for defining warning thresholds. For skin rash, the discriminatory power for the area underneath the curve (AUC) was poor (AUC = 0.522 for the pregnant cohort, and AUC = 0.610 for the postpartum cohort) and the ROC analysis was not significant for skin rash in the pregnant cohort, indicating that thresholds derived from the skin rash heat strain symptom are less suitable compared to the other symptoms. Furthermore, skin rash, more specifically Miliaria, is a skin condition that results from obstruction of sweat glands, causing sweat to build up under the skin and form a rash^29^. Although high heat and humidity promote excessive sweating and increase the risk of miliaria, its development is also influenced by factors such as clothing-related skin blocking, hygiene practices and individual skin susceptibility^30^. Therefore, skin rash may demonstrate a weaker and less direct relationship with ambient heat exposure than other heat strain symptoms, which may explain its lower discriminatory performance in the threshold analysis. Thirst, on the other hand, is strongly influenced by individual fluid intake and differed substantially between groups. From the interaction analysis, we identified dizziness to be the only heat strain symptom that significantly differed across the pregnant and postpartum cohorts. Research shows that pregnancy itself induces substantial cardiovascular adaptations to support the foetus, such as changes in blood pressure and redistributions in blood flow^31^. During heat exposure, increased skin blood flow required for heat dissipation, combined with fluid losses through sweating, can reduce central blood volume^32^. These competing circulatory demands may place additional strain on cardiovascular regulation and contribute to susceptibility to dizziness^33^, particularly among pregnant women. For postpartum women who are working and physically active, heat exposure may increase the risk of dizziness as blood flow competes between supplying the working muscles and skin for heat dissipation. In addition, fluid losses through sweating can further reduce blood volume^34^. These combined effects may reduce central circulation and the ability to maintain blood pressure, potentially contributing to feelings of dizziness. Nevertheless, the higher prevalence of symptoms in the postpartum cohort may be reflected by varying occupational and outdoor exposures. Postpartum women in this study spent more time outdoors and performing heavy work. Furthermore, time spent on heavy work was identified as a significant covariate in the dizziness association. Exhaustion and sleep disturbances showed more stable heat responses across both pregnant and postpartum women and may therefore be a more appropriate indicator for heat-strain-based thresholds. Finally, the exposure-response curve could be used as a way of personalising heat warnings: providing guidance for users to select symptom-specific or risk-specific alert levels based on WBGT. This personalised approach would provide flexibility and may better accommodate individual heat sensitivity and work conditions. Nevertheless, it is important to note that as the app was designed to encourage behavioural adaptation, participants were expected to take preventive action in response to alerts. As a result, reduced symptom severity may reflect successful behaviour change rather than an absence of risk, which may attenuate observed associations.

Heat strain may occur when the body’s heat gain exceeds its capacity for heat loss, resulting in an elevated core temperature^35^. Heat strain symptoms can appear before detectable hyperthermia and may therefore be underreported. Hot conditions are consistently linked to higher exhaustion among the general population^6^, a pattern that our study confirms among pregnant and postpartum individuals. Given that untreated heat strain can escalate to serious heat illnesses^25^, and maternal hyperthermia (≥39°C) poses risk during pregnancy^36^, understanding symptom patterns and early heat strain signals such as sweating or exhaustion and dizziness is critical for preventive action. Effective EWS require heat thresholds that reflect when adverse effects begin, especially for vulnerable groups. Currently, South Africa’s heat warnings are based on temperature-fixed thresholds for large cities, not heat-health outcomes^37^. Mortality-based thresholds are common^38^, but morbidity precedes mortality and physiological heat strain occurs even earlier. Our study addressed this gap by identifying thresholds based on early self-reported heat strain symptoms. Furthermore, epidemiological thresholds are often based on heterogeneous samples, including sex, age group, and geographical location, making them less accurate and actionable^38,39^. They often use simple air temperature metrics, limiting accuracy^6^. Our focus on a physiologically similar group and using WBGT may improve the contextual relevance of the identified thresholds, although further validation is needed. Thresholds should, however, be interpreted as indicative guidelines that vary with individual characteristics and are typically lower for less heat-tolerant populations^25,40^, such as pregnant and postpartum women.

Heat-related health risks driven by climate change can be mitigated through adaptation strategies such as EWS with well-defined thresholds^39^. Evidence-based thresholds are central to EWS and can trigger preventive actions by both the public and authorities when exposure is high^39^. The often-cited WBGT threshold of 35 °C, which represents the upper limit of human heat tolerance, assumes ideal physiological and environmental conditions that are unsuitable for vulnerable populations^25^ like pregnant women in real-world low- and middle-income countries (LMIC) settings. Statistically significant heat thresholds are important for advancing research, but may not be practical in real-world situations; if set too low, they may trigger excessive warnings and reduce the effectiveness of early warning systems^38^. A systematic review of controlled exercise and heat exposure studies during pregnancy reported that pregnant women generally remained below the proposed teratogenic core temperature threshold of 39 °C under specific tested conditions and suggested that protective thermoregulatory responses may increase as pregnancy progresses. The authors discussed that smaller increases in core temperature in later pregnancy stages may be attributable to biophysical changes associated with increasing body mass and surface area rather than altered physiological thermoregulatory control mechanisms^36^. In contrast to the controlled experimental studies included in the systematic review, our approach aimed to identify heat thresholds based on real-world exposure and symptom reporting, which may more closely reflect lived conditions but also incorporates unavoidable additional variability. Our findings indicate that temperature thresholds associated with heat strain symptoms under real-world environmental conditions vary substantially depending on symptom type, environmental condition and individual parameters. We found that heat thresholds of 21.19 °C to 26.16 °C WBGT predict increased heat strain in pregnant and postpartum women. These values may offer practical guidance for public health policy and early warning systems in similar climates. Cheng et al., 2019 describe two distinct ways of selecting heat thresholds: the thresholds observed through increases in heat-related morbidity and mortality, and those determined for issuing public warnings^39^. They note that if thresholds are determined merely following heat-health adversities data, warnings will be rather low and may lead to excessive warnings and warning fatigue^39^. Thus, lower thresholds identified in our study, e.g., the 18.53 °C for skin rash amongst the postpartum population, are likely too sensitive for operational warnings, while higher thresholds, such as those identified for dizziness (25.07 °C and 26.16 °C respectively), or sleep disturbance (23.00 °C and 25.07 °C, respectively) may be more suitable. These estimates are consistent with recent work that reevaluates the upper WBGT limits for safe activity to range between 25 °C and 31 °C depending on exact air temperature and humidity^40,41^. According to a meta-analysis, 15% of heat-exposed workers develop acute kidney injury, and 35% show increased heat strain after a single shift at WBGT > 22°C WBGT for heavy labour and 24.8 °C WBGT for most tasks^28^. Given our participants’ substantial outdoor and heavy work exposure, these thresholds are relevant.

The need is particularly acute in LMICs, which face disproportionate climate impacts yet remain significantly underrepresented in heat-health research^42^. Many regions are projected to become increasingly unliveable due to increases in heat, with proportions of liveable land shrinking dramatically under high-emission scenarios^25^. For many pregnant, low-wage individuals in LMIC, multiple vulnerability factors intersect: higher heat exposure due to hotter climates^6^, reduced heat tolerance due to pregnancy^43^ and sometimes outdoor work^6^. Many pregnant and postpartum women continue working late into pregnancy and resume work shortly after birth^11^, yet occupational heat strain threatens both health and productivity^28^. Furthermore, regular pregnancy checks are essential for maternal health and although South African rates of antenatal care visits are high^44^, socially marginalised women and those with unintended pregnancies are less likely to seek care^43^. Heat exposure for pregnant and postpartum women is therefore shaped by social and economic factors, gendered divisions of labour, informal or insecure work, and socioeconomic uncertainty, which often makes it difficult to rest, hydrate, and avoid peak heat periods. These intersecting factors create patterned heat vulnerabilities that cannot be addressed through individual-level warnings alone. This highlights the need for responses beyond individual-level warning systems, including workplace protection, improved access to antenatal care, and housing and community-level heat adaptation strategies that consider the needs of pregnant and postpartum individuals.

Behavioural adaptation, a key coping mechanism for heat, is often constrained in LMICs^25^. Poorly insulated housing and limited access to affordable cooling prolong indoor and outdoor heat exposure^6^. In our study, 51% of participants reported that their homes were hotter indoors than outdoors. Individuals with lower socioeconomic status are more likely to live in such housing^45^, and since these marginalised groups were underrepresented in our sample, the estimated heat-strain thresholds are likely overestimated, suggesting that thresholds would be lower if more individuals experiencing hotter indoor conditions had been included. Air conditioning remains inaccessible for many due to cost and infrastructure limitations^6,38^. Faurie et al., 2022 similarly found that low-income groups without air conditioning are at higher risk of heat strain^42^. Geographically tailored thresholds are essential, particularly in densely populated regions where medical resources are limited and self-protection is important for adaptation^39^. Such thresholds would ideally need to account for regional climatic conditions, indoor heat exposure and physical load (e.g. through varying occupations), rather than relying on a single, universal threshold. Our study contributes to further understanding of heat strain symptoms in pregnant and postpartum individuals and may help to inform the identification of South Africa- specific thresholds for this population.

Although this study has contributed to the field of heat and maternal health by identifying heat thresholds based on heat strain symptoms, this study has several limitations. First, the small sample size and convenience sampling introduce selection bias. This may have been further amplified by recruiting participants through antenatal clinics and following them via app engagement, likely underrepresenting more marginalised individuals. In addition, selection bias is likely particularly pronounced due to the exclusion of individuals without smartphone access, who are often among the most socioeconomically disadvantaged. Therefore, the participants are not representative of those women both at the highest and lowest ends of the socioeconomic spectrum. Second, and as a result, the use of convenience sampling limits generalisability, and the findings reflect a specific subgroup of the South African population which cannot be generalised to all women or all regions of the country. Third, the study relied heavily on self-reported data, which introduces response and desirability bias. Due to enrolment procedures and regular heat-warning adaptation messaging, participants were likely aware of the project ambitions and goals, and misclassification of symptom under- or over- reporting cannot be ruled out. Fourth, indoor temperatures, an important parameter when assessing the health impacts of heat exposure^43,46^, were not considered in this study, despite half of participants reporting hotter indoor conditions. The questionnaires, although comprehensive, were not validated, and their length required reducing detail on key domains such as thermo-physiological responses, cooling behaviours, cognition and concentration. For comparability between cohorts, gestational age was not included as a covariate, despite its known influences on heat responses^47^. Also, socioeconomic status was not assessed beyond housing type and employment. Fifth, the absence of a validation cohort or external replication may affect the robustness and generalisability of the findings. Sixth, seasonal acclimatisation was not assessed, although evidence suggests that heat-related risks may vary across the season, particularly before acclimatisation occurs^48^.

This study applies multiple heat indicators (WBGT, UTCI and Tmax), which limits direct comparability across analyses. WBGT served as the primary exposure metric for most heat-related questions, with Tmax applied simultaneously as a comparative measure. UTCI was used separately for a single additional question tied to the in-app data source. Future studies would benefit from adopting a consistent heat metric throughout to improve coherence. Moreover, the heat strain outcomes in this study are based on a 2-week recall period, which captures events over a broad time window and limits our ability to assess precise temporal relationships or lagged effects. In addition, the wetness measure used in this study was intended to capture participants’ subjective perception of skin wetness rather than a strict thermophysiological measure. However, closer alignment with established thermophysiological concepts, such as skin wetness^49^, may have been beneficial. Baseline variability in wetness sensation suggests that participants differed widely in how likely they were to report feeling sweaty, given the same temperature. This is supported by previous literature, which highlights that sweating varies both between individuals and within individuals depending on factors such as surface area to mass ratio, fitness level and acclimatisation status^50^. This suggests that wetness sensation differs widely across participants, making it difficult to interpret overall tendencies. Furthermore, in-app sweat data could not be disaggregated between pregnant and postpartum users due to anonymity. Also, finding no association between dehydration (USG) and heat measured using WBGTmax was likely due to non-standardised data collection and should be standardised to early mornings, following routine USG protocols, in future studies. Lastly, the heat strain symptoms assessed in this study were rather crude.

Although WBGT is a widely used integrated heat stress metric, it only captures individual physiological variation or differences in activities and clothing through crude estimates. Participants’ daily heavy work exposure varied considerably, potentially affecting the accuracy of WBGT-based thresholds. UTCI addresses some of these limitations through a more advanced thermophysiological model^35^, but was only used for the wetness sensation analyses. Although acknowledging their importance in the impact of heat on health, we did not account for heatwave characteristics, including frequency, severity and duration. Furthermore, temperature was modelled as a continuous variable, with effect estimates expressed per 1 °C increase. However, this assumes linearity and may not fully capture potential non-linear relationships. Lastly, the smartphone application served both as a data collection tool and an intervention through its heat warnings and behavioural advice. Warnings were designed to increase awareness of heat-related symptoms, thereby influencing symptom perception and self-reporting. Simultaneously, warnings encouraged protective behaviours that reduced heat exposure or risk of adverse heat consequences. These mechanisms could both lead to higher self-reported symptom frequencies on hot days, yet simultaneously also mitigate heat-related symptoms, thereby attenuating estimated associations between ambient heat exposure and symptoms. Accordingly, findings should be interpreted as representing heat-symptom relationships observed in the context of app-based warnings rather than a purely observational setting. Warnings issued through the EWS may have influenced the behaviour of participants, potentially leading to an underestimation of the harmful effects of heat.

Continued global warming will threaten the survival of humans and other species that are unable to avoid, migrate or adapt to extreme or prolonged heat stress^35^. Effective heat EWS with appropriate indicators and thresholds are therefore increasingly critical to reduce health risks^28,39^. It may be beneficial to consider whether stakeholder engagement could support the identification of context-appropriate heat thresholds. Heat-health associations grounded in physiological models enhance our understanding of how humans can live and work in a hotter future, moving beyond heat associations focusing on survival and death^25^. We agree with Faurie et al. (2022) that policy- and decision-makers should be better informed to understand heat risks for vulnerable groups and target preventive strategies, such as EWS^42^. Existing heat-health EWSs vary widely in indicators and threshold methods^38^. Integrating human physiological principles into climate and health data can strengthen assessments of individual heat exposure and strain^6^, enabling more targeted and effective EWSs for vulnerable populations. Research emphasises the need for increased public knowledge, adaptive behaviours, health system preparedness and equitable access to cooling^42^. For health systems, these findings also imply that routine maternal care may need adaptation, such as scheduling ANC visits to cooler hours, improving ventilation in clinics and integrating heat counselling into ANC protocols, to ultimately reduce heat strain among both patients and their providers. EWS should provide timely care and reduce heat adversities for mothers and infants^43^.

This study demonstrates a clear association between heat exposure and multiple heat strain symptoms, including sleep disturbances, dizziness, thirst, exhaustion and wetness sensation, in pregnant and postpartum women. We identified symptom-specific heat thresholds corresponding to the optimal ROC-derived cutoff for both cohorts. Effective heat thresholds should satisfy both scientific and policy criteria, reflected by statistically significant, yet meaningful health effects^38^. Future studies should employ distributed lag non-linear models (DLNM) as a more comprehensive statistical approach that could capture non-linear and delayed exposure-response relationships between heat and health outcomes. Furthermore, it is important to note that this study was conducted under moderate heat stress conditions (WBGT range: 16.0–28.8 °C), and future research is needed to assess whether these findings generalise to higher levels of heat exposure. Indoor temperature monitoring should also be addressed in future studies, as it is an important component of prolonged heat exposure and captures important individual differences in exposure. Furthermore, as heat risk is not considered the same throughout pregnancy^51,52^, future studies should integrate gestational age into their analysis, making it trimester-specific. In addition to USG, additional physiological measures such as core body temperature and heart rate variability could be addressed to strengthen the mechanistic understanding. As global temperatures rise, there is an urgent need to develop new heat thresholds to protect vulnerable groups, including pregnant and postpartum women. While our study takes a first step, more research across diverse settings is needed to protect mothers and their infants from heat adversities. In line with the SDGs and principles of “leaving no one behind”^53^, we must prioritise high-exposure, physiologically vulnerable subgroups to protect those who need it the most in future heat-health research and policy. Our findings provide evidence that can support policymakers in setting context-appropriate heat thresholds and guiding targeted heat-health interventions for this population. Nevertheless, future studies should aim to validate these findings in independent LMIC cohorts before considering their application to policy or practice. We hope this work contributes to improved protection for pregnant and postpartum women and reduces heat-related health impacts in a warming world.

## Methods

### Study setting

The study was conducted between October 2024 and August 2025 at a Community Health Centre (CHC) in Tshwane District, Johannesburg, Gauteng Province, South Africa. Located in an arid climate zone (BWh) according to Köppen-Geiger climate classifications^54^, the hottest months are typically December and January, with mean monthly maximum temperature ranging from 26.9 °C to 27.4 °C [40.3–52.1% RH], extracted from hourly outdoor weather data values from the Copernicus Climate Data Storage for the period 2019 to 2024 at the latitudes corresponding to the location of the clinics^55^. The district is predominantly urban but includes extensive informal settlements, where most households lack cooling infrastructure.

### Questionnaires and in-app survey

**Fig. 3:**
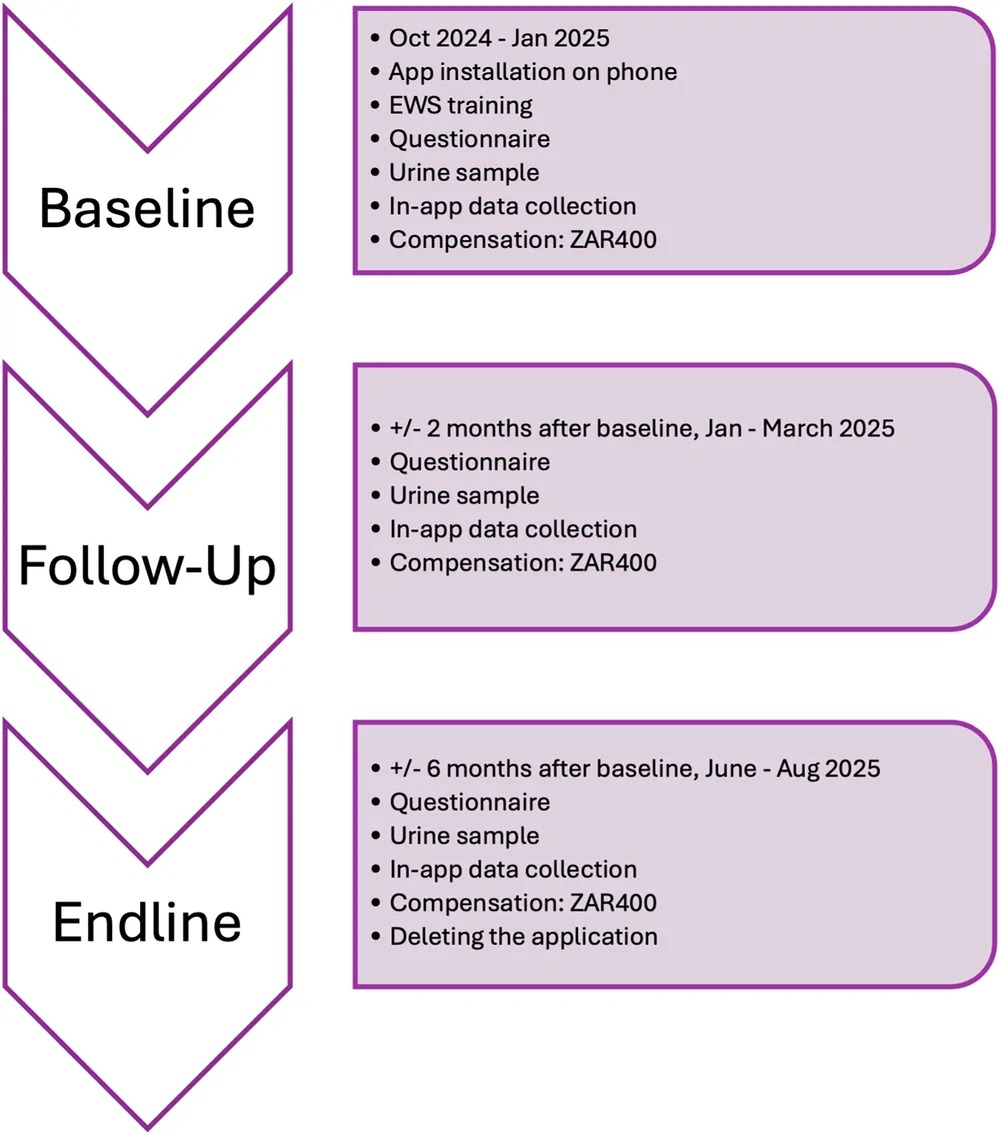
Participant flow in the HIGH Horizon study. Participation of pregnant and postpartum women in the HIGH Horizon study, including baseline, follow-up and endline visit.

Data were collected using both self-reported and measured health and environmental data through two primary sources: structured questionnaires and in-app data surveys. Structured questionnaires were interviewer-administered at three time points: baseline, follow-up, and endline (Fig. 3). Follow-up visits were conducted at two and six months after enrolment, each session lasting around 60 minutes. These visits included questionnaire administration and urine sample collection. Responses were collected on paper and captured into a REDCap database^56^. Data quality checks were performed by study staff to ensure data were accurately captured from paper questionnaires into the REDCap database. The questionnaires were slightly adapted across the three timepoints to avoid repetition on demographic variables but were designed to gather detailed information on participants’ socio-demographic profiles, medical and obstetric histories, knowledge about and behaviours related to heat risks and physiological responses and actions taken by participants based on the recommendations of the MotherHeat Alert app. However, they were not validated instruments. Separate from the questionnaires, throughout the study period, participants could voluntarily complete a brief seven-question in-app survey designed to gather real-time data on heat-related symptoms and perceptions. Participants could complete the survey as frequently as they wished and the responses were directly linked to the environmental factors such as the local, current UTCI.

### Heat exposure

Questionnaire data included the date, time and spatial coordinates of questionnaire completion. This information was used to extract weather data from ERA5 hourly data, including air temperature, humidity, wind speed and solar radiation from the Copernicus Climate Data Store^57^. The environmental ERA5 parameters were converted into WBGT data using Liljegren’s Model^58^, as a more comprehensive heat stress measure, compared to air temperature alone. WBGT incorporates temperature, humidity, solar radiation, and wind, providing a multidimensional assessment of heat burden^38,39^. It is considered one of the most sensitive heat stress indicators^31,34^, particularly for vulnerable groups^34^. Relying only on plain air temperature risks missing heat stress events and undermining EWS effectiveness^39^. Importantly, nonetheless, ERA5 reanalysis data may not capture microclimate variability and have been shown to underestimate the true heat exposure of informal settlements in hot areas^59^. To analyse the effect of heat exposure on heat strain, we calculated the 14-day average of the daily maximum air temperature (Tmax) and maximum wet-bulb globe temperature (WBGTmax) for the two-week period prior to the completion of the questionnaire. Daily maximum rather than mean temperature was used to capture peak heat stress conditions, as daily mean temperatures may mask periods of high exposure through low nighttime values. A 14-day exposure window was selected to align with the 2-week recall period used for heat strain symptom questions, to ensure that the exposure metric corresponded to the timeframe over which symptoms were reported. For USG data, daily maximum WBGT and daily maximum temperature were used to analyse levels of dehydration on the day the urine sample was taken. Throughout this study, WBGTmax was used as the main heat indicator, whereas separate Tmax results can be found in the supplementary material. Finally, this study did not measure indoor temperature.

Heat strain data from the questionnaire included the self-reported occurrence in the past two weeks of skin rash, sleep disturbance, dizziness, intense thirst and exhaustion. Furthermore, the in-app survey question assessing the level of wetness on skin due to sweating was analysed according to the directly displayed in-app Universal Thermal Climate Index (UTCI) current value. For more detailed information on the items and their response options (Table S17). UTCI was chosen as a heat stress indicator for the in-app survey responses, as the MotherHeat Alert warnings issued rely on UTCI data. This way, the in-app survey responses directly corresponded with UTCI temperature. Similarly to WBGT, UTCI is a more comprehensive heat stress index that incorporates both clothing and physical activity estimates in the prediction^60^. Therefore, WBGT was used as a heat stress indicator for baseline, follow-up and endline results, whereas UTCI was used for in-app survey responses.

### Urine specific gravity

Urine specific gravity (USG) was assessed as a biological heat strain indicator assessing levels of dehydration at the baseline, follow up and endline visits. The participants provided mid-stream urine samples, and USG was measured using the PAL-10S digital optical refractometer on site. The testing was carried out by trained research staff. Unlike standard procedures in which USG samples are collected during first morning urination, urine samples in this study were collected at a convenient time, typically at the end of questionnaire completion or during antenatal visits. For most participants, this occurred in the early afternoon. As the timing of urine collection was not standardised, the interpretation of hydration status is substantially limited, and likely reflects a more acute hydration status rather than chronic heat strain. Following testing, all urine specimens were discarded, according to on-site biological waste management protocol.

### Study participants

Participants were offered participation, enroled using convenience sampling and followed up for six months. Two participant cohorts were enroled:

1. Pregnant individuals who were confirmed pregnant and whose second trimester had been verified by a registered health worker.
2. Postpartum individuals with live-born infants aged 0 to 9 months.

This design included a wide range of participants, following up pregnant women in their second trimesters to women 15 months postpartum, considering the six-month duration of the study. Eligibility criteria included: being 18 years or older, owning a smartphone, intending to live in the study area during the six-month follow-up period and providing written informed consent for participation. Clinical trial number: not applicable.

### Sampling and recruitment

Participants were recruited from the antenatal and childhood immunisation clinics at a large Community Health Centre (CHC). Recruitment rates reached a total of 280 potential participants for entry into the study, of which 209 (74.6%) signed the informed consent form. Of those not entered, 26 (9.3%) did not have a compatible smartphone, 14 (5.0%) were not planning on remaining in the area for 6 months and 51 (18.2%) were not willing and/or not able to provide informed consent (note that numbers are not mutually exclusive). Ultimately, 201 participants were fully enroled, upon which the analyses in this study are based. Recruitment materials, including flyers and posters with brief study information and contact details, were displayed in the maternity unit and other health wards. English, Ndebele, Sepedi, Setswana, Zulu and seSotho were the languages spoken throughout recruitment. Enrolment visits lasted approximately 60-90 minutes, and included obtaining informed consent, administering the baseline questionnaire assessing height and weight and collecting urine samples for osmolality and specific gravity. Participants also received an additional 30-minute training session on installing and configuring the MotherHeat Alert app, an app-based Early Warning System (EWS) specifically designed to warn pregnant and postpartum individuals of upcoming heat exposure. The app is described in more detail in section 4.9. User profiles were set up with the participants within the app, including key demographic information such as age, body weight and height.

### Statistical analysis

For statistical analyses, heat-related symptom variables were treated as binary outcomes (yes = 1, no=0), and weather data as continuous. Responses of “don’t know” were recoded as missing. A binary logistic regression using General Estimating Equations (GEE) with an exchangeable working correlation structure was applied to estimate the probability that a specific heat symptom (e.g., skin rash) occurred due to the independent variables, WBGTmax and Tmax. As multiple heat strain symptoms were tested, we corrected for multiple comparisons using the Bonferroni correction. For USG, we used a non-parametric Spearman’s rank correlation to examine the association between WBGTmax and Tmax. For the in-app wetness sensation question, a multinomial generalised linear mixed model (GLMM) was applied to model the likelihood of reporting the respective response options (Table S17). Furthermore, covariates were assessed for potential multicollinearity by conducting a standard multiple linear regression using the abovementioned covariates as predictors. Tolerance statistics and variance inflation factor (VIF) statistics were assessed. All analyses were carried out with SPSS version 30.0. Pregnant and postpartum cohorts were stratified to derive cohort-specific heat thresholds. In addition, pooled interaction analyses including cohort as a covariate were conducted to evaluate whether significant differences between groups existed. Finally, receiver operating characteristic (ROC) analysis was conducted to identify data-driven heat thresholds associated with the respective heat strain symptoms. These thresholds were determined using Youden’s index.

### Covariates

Based on previous knowledge, covariates were selected a priori, as they were well-documented risk factors of adverse pregnancy outcomes^61–63^, including general health^64^, maternal age^65^, and parity^66^. Additional covariates, including housing type, hours spent on heavy work, and whether it is hotter indoors (assessed through self-reports on perceived heat outdoors vs indoors), were incorporated to account for external factors potentially influencing individuals’ heat balance^67,68^. For instance, housing type can contribute to a hotter indoor environment; in the study area, many participants reside in informal settlements where homes are typically low thermal mass, built with lightweight corrugated steel sheets and have minimal windows. In contrast, time spent on heavy work captures the metabolic heat production in addition to the ambient heat exposure. These two covariates were evaluated along with other potential variables and identified as most relevant. Nevertheless, residual confounding remains likely. More information on how covariates were assessed is in Table S9.

### The mobile application intervention: MotherHeat Alert

MotherHeat Alert is a mobile application that functions as an early warning system (EWS) and is available for both Android and iOS devices to be applicable to users across different end devices; however, it is not yet open for download to all. It delivers real-time, localised heat alerts alongside specific recommendations tailored to pregnant and postpartum users, to facilitate individualised protective actions. The system integrates environmental data, estimated values of the Universal Thermal Climate Index (UTCI), with individual user characteristics such as age and body mass index (BMI). A pregnancy or postpartum profile can be set up, which determines the timing of the alerts and content of the recommendations. The EWS directly incorporates local weather data from OpenWeatherMap, based on the location (longitude, latitude) and timestamp and automatically displays UTCI in the app. Daily maximum UTCI was used to trigger heat risk warnings in the MotherHeat Alert if maximum UTCI exceeded the EWS threshold. For instance, if a warning threshold is set to 27 °C UTCI, and the UTCI temperature exceeds this threshold, users are notified with a MotherHeat Alert warning. The EWS was designed to increase awareness of heat-related health risks and encourage adaptive behaviours, such as directly cooling the body, drinking water, resting or limiting sun exposure during warning periods. Smartphone penetration in South Africa was estimated at 91% and the Gauteng province, including Tshwane district, has full 4 G mobile coverage. Most eligible participants were therefore expected to have access to a smartphone.

### Regulatory approval and confidentiality

Ethical approval was obtained from the University of the Witwatersrand Human Research Ethics Committee (HREC) in South Africa; ethics number 240606. District approval was also obtained to conduct the study in a Tshwane health facility. The study was registered with the South African National Clinical Trial Registry (DOH-27-032025-5623). All participants provided written informed consent prior to enrolment. Data were either fully anonymised or pseudonymised, participants were assigned a unique identification number and physical copies with identifiable information were stored in securely locked cabinets. Electronic data could only be accessed through password-protected servers with two-factor authentication. Participants received a reimbursement of ZAR400 for each study visit. All staff involved in the conduct of the project completed the following training and all research was undertaken according to these standards: International conference on Harmonisation (ICH)/ WHO Good Clinical Practice (ICH GCP with E6 (R3) updates) underpinned by the principles of the Declaration of Helsinki including guidance on various ethics-related processes and procedures and locally prescribed South African Department of Health Ethics in Health Research modules; and Ethics and Human Subject Protection with modules in Research Ethics and Informed Consent.

## Supplementary information

Supplementary information

## Data Availability

The datasets generated and analysed during the current study are not publicly available due to sensitive health data and participant confidentiality but are available from the corresponding author on reasonable request.
