# Supplementary material for: Effects of heat exposure on self-reported heat strain symptoms among pregnant and postpartum women in South Africa
Source: NPJ Digit Public Health. 2026 Sep 13;1(1):33. doi: 10.1038/s44482-026-00036-4 (PMC13572200; doi:10.1038/s44482-026-00036-4)
Supplement: Supplementary file 1 [file 44482_2026_36_MOESM1_ESM.docx]

**Supplementary Information**

***Table S1****. Education and employment status of participants. Note that employment status was not mutually exclusive.*

| **Descriptive statistics of participants** | | | | |
| --- | --- | --- | --- | --- |
|  |  |  | **N** | **Percent** |
| **Education** | Completed primary school | pregnant | 1 | .5 |
|  |  | postpartum | 0 | 0 |
|  | Some high / secondary school | pregnant | 17 | 17.6 |
|  |  | postpartum | 19 | 18.8 |
|  | Completed high/secondary school | pregnant | 54 | 57.1 |
|  |  | postpartum | 63 | 62.4 |
|  | Some tertiary / university / college education | pregnant | 20 | 13.7 |
|  |  | postpartum | 8 | 7.9 |
|  | Completed tertiary / university / college education | pregnant | 8 | 9.3 |
|  |  | postpartum | 11 | 10.9 |
| **Employment** | Employed full-time | pregnant | 35 | 34.3 |
|  |  | postpartum | 28 | 27.7 |
|  | Employed part-time | pregnant | 10 | 9.8 |
|  |  | postpartum | 3 | 3.0 |
|  | Self-employed | pregnant | 7 | 6.9 |
|  |  | postpartum | 0 | 0 |
|  | Not employed but looking for work | pregnant | 42 | 41.2 |
|  |  | postpartum | 58 | 57.4 |
|  | Not employed and not able to work | pregnant | 0 | 0 |
|  |  | postpartum | 7 | 6.9 |
|  | Not employed and not looking for work | pregnant | 2 | 2.0 |
|  |  | postpartum | 0 | 0 |
|  | Homemaker | pregnant | 5 | 4.9 |
|  |  | postpartum | 5 | 4.9 |
|  | Student | pregnant | 1 | 1.0 |
|  |  | postpartum | 0 | 0 |

***Table S2****. Health status of participants. Self-rated general health status of the participant, and chronic conditions. No participant indicated to diagnosed with: Dyslipidaemia, Tuberculosis (TB), Autoimmune disease (Lupus, rheumatoid arthritis), Osteoarthritis, Heart disease, Stroke, Epilepsy, Depression / Anxiety or other mental illness or another chronic illness in general. COPD stands for Chronic Obstructive Pulmonary Disease.*

| Health Status of Participants | | | | |
| --- | --- | --- | --- | --- |
| **General health today** |  |  | **N** | **Percent** |
|  | Very good | pregnant | 36 | 36 |
|  |  | postpartum | 30 | 29.7 |
|  | Good | pregnant | 55 | 55 |
|  |  | postpartum | 62 | 61.4 |
|  | Neither good nor poor | pregnant | 8 | 8 |
|  |  | postpartum | 8 | 7.9 |
|  | Poor | pregnant | 1 | 1 |
|  |  | postpartum | 1 | 1 |
|  | Very poor | pregnant | 0 | 0 |
|  |  | postpartum | 0 | 0 |
|  | Total |  | 201 | 100 |
| **Chronic condition** | **Yes** | pregnant | 9 | 9 |
|  |  | postpartum | 20 | 19.8 |
|  | *Hypertension* | pregnant | 1 | 1 |
|  |  | postpartum | 3 | 3 |
|  | *Diabetes* | pregnant | 0 | 0 |
|  |  | postpartum | 1 | 1 |
|  | *Asthma/COPD* | pregnant | 0 | 0 |
|  |  | postpartum | 1 | 1 |
|  | *HIV* | pregnant | 8 | 8 |
|  |  | postpartum | 15 | 14.9 |
|  | **No** | pregnant | 91 | 91 |
|  |  | postpartum | 81 | 80.2 |

***Table S3****. Pregnancy history: gravidity and parity status.*

| **Pregnancy History** | | | | | |
| --- | --- | --- | --- | --- | --- |
|  |  | **Gravidity** | | **Parity** | |
|  |  | **N** | **Percent** | **N** | **Percent** |
| 0 | pregnant | 0 | 0 | 26 | 26 |
|  | postpartum | 0 | 0 | 0 | 0 |
| 1 | pregnant | 20 | 20 | 36 | 36 |
|  | postpartum | 23 | 22.8 | 29 | 28.7 |
| 2 | pregnant | 33 | 33 | 23 | 23 |
|  | postpartum | 38 | 37.6 | 39 | 38.6 |
| 3 | pregnant | 30 | 30 | 9 | 9 |
|  | postpartum | 24 | 23.8 | 26 | 25.7 |
| 4 | pregnant | 9 | 9 | 5 | 5 |
|  | postpartum | 12 | 11.9 | 5 | 5.0 |
| 5 | pregnant | 7 | 7 | 1 | 1 |
|  | postpartum | 2 | 2.0 | 2 | 2.0 |
| 6 | pregnant | 1 | 1 | 0 | 0 |
|  | postpartum | 0 | 0 | 0 | 0 |
| 7 | pregnant | 0 | 0 | 0 | 0 |
|  | postpartum | 1 | 1.0 | 0 | 0 |
| 9 | pregnant | 0 | 0 | 0 | 0 |
|  | postpartum | 1 | 1.0 | 0 | 0 |
| Total |  | 201 | 100 | 201 | 100 |

***Table S4****. Pregnancy characteristics, including gestational age at delivery and singleton status.*

| **Pregnancy characteristics: Delivery and Singleton Status** | | | |
| --- | --- | --- | --- |
|  |  | **N** | **Percent** |
| **Delivery** | When it was due (37-42 weeks) | 87 | 91.6 |
|  | One month before due date (<37 weeks) | 7 | 7.4 |
|  | After due date (>42 weeks) | 1 | 1.1 |
|  | Total | 95 | 100 |
| **Singleton pregnancy** | Yes | 88 | 88 |
|  | No | 5 | 5 |
|  | Don’t know | 7 | 7 |
|  | Total | 100 | 100 |

***Table S5****. Housing characteristics. Participants did not select any of the following other answer options for type of roof: asbestos, plastic, roofing shingles, no roof, thatch/palm leaf, rustic mat, palm/bamboo, wood planks, cardboard, metal, don’t know, other. ** e.g. informal squatter settlement on a farm.

| **Type of roof** | Cement | pregnant | 1 | 1 |
| --- | --- | --- | --- | --- |
|  |  | postpartum | 0 | 0 |
|  | Wood | pregnant | 23 | 23 |
|  |  | postpartum | 26 | 25.7 |
|  | Tiles | pregnant | 1 | 1.0 |
|  |  | postpartum | 1 | 1.0 |
|  | Iron sheet / corrugated metal | pregnant | 75 | 75 |
|  |  | postpartum | 74 | 73.3 |
|  | Total |  | 201 |  |
| **Indoor heat** | Indoor is cooler | pregnant | 38 | 38 |
|  |  | postpartum | 46 | 45.5 |
|  | Indoor is hotter | pregnant | 49 | 49 |
|  |  | postpartum | 53 | 52.5 |
|  | Just as hot inside as outside | pregnant | 10 | 10 |
|  |  | postpartum | 2 | 2.0 |
|  | Don’t know | pregnant | 3 | 1.5 |
|  |  | postpartum | 0 | 0 |
|  | Total |  | 201 |  |

***Table S6****. Descriptive statistics of heat-related symptoms gathered through interview-administered questionnaires at baseline, follow up and endline, stratified amongst observations reported by pregnant individuals (N = 300) and postpartum individuals (N = 303).*

|  |  |  | **N** | **Valid Percent** |
| --- | --- | --- | --- | --- |
| **Skin Rash** | no | pregnant | 225 | 86.5 |
|  |  | postpartum | 229 | 86.1 |
|  | yes | pregnant | 35 | 13.5 |
|  |  | postpartum | 37 | 13.9 |
|  | missing | pregnant | 40 | na |
|  |  | postpartum | 37 | na |
|  | total |  | 603 |  |
| **Sleep** | no | pregnant | 136 | 51.7 |
|  |  | postpartum | 153 | 57.5 |
|  | yes | pregnant | 127 | 48.3 |
|  |  | postpartum | 113 | 42.5 |
|  | missing | pregnant | 37 | na |
|  |  | postpartum | 37 | na |
|  | total |  | 603 |  |
| **Dizziness** | no | pregnant | 181 | 69.1 |
|  |  | postpartum | 194 | 73.5 |
|  | yes | pregnant | 81 | 30.9 |
|  |  | postpartum | 70 | 26.5 |
|  | missing | pregnant | 38 | na |
|  |  | postpartum | 39 | na |
|  | total |  | 603 |  |
| **Thirst** | no | pregnant | 97 | 37 |
|  |  | postpartum | 112 | 42.4 |
|  | yes | pregnant | 165 | 63 |
|  |  | postpartum | 152 | 57.6 |
|  | missing | pregnant | 38 | na |
|  |  | postpartum | 39 | na |
|  | total |  | 603 | 100 |
| **Exhaustion** | no | pregnant | 108 | 41.2 |
|  |  | postpartum | 128 | 48.5 |
|  | yes | pregnant | 154 | 58.8 |
|  |  | postpartum | 136 | 51.5 |
|  | missing | pregnant | 38 | na |
|  |  | postpartum | 39 | na |
|  | total |  | 603 |  |

***Table S7****. In-app question, descriptive statistics of number of answers of the different sweat categories.*

| **Please rate your current level of wetness due to sweating** | | |
| --- | --- | --- |
|  | **N** | **Percent** |
| dry | 3865 | 59.5 |
| a little wet | 1111 | 17.1 |
| wet | 1213 | 18.7 |
| extremely wet | 299 | 4.6 |
| missing | 6 | 0.2 |
| total | 6494 | 100 |

***Table S8****. Collinearity statistics indicating no concern for collinearity between variables in either cohort. Tolerance <0.10 problematic, VIF >5: moderate, >10: severe.*

| **Covariates** | **Cohort** | **Tolerance** | **VIF** |
| --- | --- | --- | --- |
| Maternal Age | pregnant | .457 | 2.187 |
|  | postpartum | .584 | 1.714 |
| Parity | pregnant | .457 | 2.188 |
|  | postpartum | .545 | 1.835 |
| General Health | pregnant | .973 | 1.027 |
|  | postpartum | .921 | 1.085 |
| Type of house | pregnant | .868 | 1.152 |
|  | postpartum | .758 | 1.318 |
| Heavy work | pregnant | .970 | 1.031 |
|  | postpartum | .981 | 1.019 |
| Hotter Indoors | pregnant | .841 | 1.190 |
|  | postpartum | .756 | 1.323 |

***Table S9****. Overall group covariate* *Wald χ² statistic with associated significance value using WBGTmax model for pregnant and postpartum cohort. The categories of all covariates were the following: Maternal age*: <20, 20-24, 25-29, 30-34, ≥35. *Parity*: 1, 2, 3, 4, 5. *General health*: very good, good, neither good nor poor, poor, very poor. *Type of house*: house or brick / concrete structure on separate strand, flat or apartment in block of flats, house, flat or room separate from main dwelling in backyard, informal dwelling or shack in backyard, informal dwelling not in backyard, room or flat which is part of main dwelling property. *Heavy work*: 0 –45 min, 46 –120 min, ≥121 min. *Hotter indoors*: indoor is cooler, indoor is hotter, just as hot inside as outside. *Some covariates were adjusted, and response options were removed or merged for analysis due to too few observations in each category, parity adjusted: 1, 2, 3, 4+, general health: no very poor, house: no* flat or apartment in block of flats or room or flat which is part of main dwelling property.

|  |  | **pregnant** | | | **postpartum** | | |
| --- | --- | --- | --- | --- | --- | --- | --- |
| **Heat Symptom** | **Covariate** | **Wald χ² (df)** | **df** | **p** | **Wald χ² (df)** | **df** | **p** |
| Skin Rash | Maternal Age | 2.246 | 3 | .523 | 1.397 | 3 | .845 |
|  | Parity | 5.794 | 4 | .215 | 1.203 | 4 | .752 |
|  | General Health | 4.156 | 2 | .125 | .221 | 2 | .896 |
|  | Type of house | 2.581 | 3 | .461 | 2.488 | 3 | .477 |
|  | Heavy work | .013 | 2 | .993 | 2.636 | 2 | .268 |
|  | Hotter Indoors | 2.999 | 2 | .223 | 469.916 | 2 | **<.001** |
| Sleep Disturbance | Maternal Age | 1.270 | 3 | .736 | 1.497 | 3 | .827 |
|  | Parity | .291 | 4 | .990 | 2.237 | 4 | .525 |
|  | General Health | .117 | 2 | .943 | 1.174 | 2 | .556 |
|  | Type of house | 3.476 | 3 | .324 | 5.825 | 3 | .120 |
|  | Heavy work | .460 | 2 | .795 | 5.057 | 2 | .080 |
|  | Hotter Indoors | .990 | 2 | .610 | .402 | 2 | .818 |
| Dizziness | Maternal Age | .068 | 3 | .995 | 1.554 | 3 | .817 |
|  | Parity | 8.761 | 4 | .067 | 3.870 | 4 | .276 |
|  | General Health | .469 | 2 | .791 | .097 | 2 | .953 |
|  | Type of house | 4.408 | 3 | .221 | 2.693 | 3 | .441 |
|  | Heavy work | .452 | 2 | .798 | 8.338 | 2 | **.015** |
|  | Hotter Indoors | 6.665 | 2 | **.036** | 408.572 | 2 | **<.001** |
| Thirst | Maternal Age | 7.266 | 3 | .064 | 7.045 | 3 | .134 |
|  | Parity | 2.884 | 4 | .577 | 3.783 | 4 | .286 |
|  | General Health | 5.093 | 2 | .078 | .478 | 2 | .788 |
|  | Type of house | 1.356 | 3 | .716 | 1.039 | 3 | .792 |
|  | Heavy work | 4.616 | 2 | .099 | .079 | 2 | .961 |
|  | Hotter Indoors | 5.771 | 2 | .056 | 4.493 | 2 | .106 |
| Exhaustion | Maternal Age | .156 | 3 | .984 | 2.161 | 3 | .706 |
|  | Parity | 4.105 | 4 | .392 | 17.576 | 4 | **<.001** |
|  | General Health | .279 | 2 | .870 | 2.337 | 2 | .311 |
|  | Type of house | 11.317 | 3 | .**010** | 8.557 | 3 | **.036** |
|  | Heavy work | 4.304 | 2 | .116 | .391 | 2 | .822 |
|  | Hotter Indoors | 1.546 | 2 | .462 | 506.823 | 2 | **<.001** |

***Table S10****. Overall group covariate* *Wald χ² statistic with associated significance value using WBGTmax model for a pooled pregnant and postpartum estimate. The categories of all covariates were the following: Maternal age*: <20, 20-24, 25-29, 30-34, ≥35. *Parity*: 1, 2, 3, 4, 5. *General health*: very good, good, neither good nor poor, poor, very poor. *Type of house*: house or brick / concrete structure on separate strand, flat or apartment in block of flats, house, flat or room separate from main dwelling in backyard, informal dwelling or shack in backyard, informal dwelling not in backyard, room or flat which is part of main dwelling property. *Heavy work*: 0 –45 min, 46 –120 min, ≥121 min. *Hotter indoors*: indoor is cooler, indoor is hotter, just as hot inside as outside. *Some covariates were adjusted, and response options were removed or merged for analysis due to too few observations in each category, parity adjusted: 1, 2, 3, 4+, general health: no very poor, house: no* flat or apartment in block of flats or room or flat which is part of main dwelling property. Cohort refers to whether participants were classified as pregnant or postpartum. Cohort was only significant for dizziness*.*

| **Heat Symptom** | **Covariate** | **Wald χ²** | **df** | **p** |
| --- | --- | --- | --- | --- |
| Skin Rash | WBGTmax | 10.991 | 1 | <.001 |
|  | Cohort | .233 | 1 | .629 |
|  | Maternal Age | .480 | 4 | .975 |
|  | Parity | 1.261 | 4 | .868 |
|  | General Health | .633 | 2 | .729 |
|  | Type of house | 4.798 | 3 | .187 |
|  | Heavy work | 1.526 | 2 | .466 |
|  | Hotter Indoors | 1.724 | 2 | .422 |
| Sleep Disturbance | WBGTmax | 87.238 | 1 | <.001 |
|  | Cohort | .935 | 1 | .334 |
|  | Maternal age | 1.776 | 4 | .777 |
|  | Parity | 1.582 | 4 | .812 |
|  | General Health | .992 | 2 | .630 |
|  | Type of house | 11.152 | 3 | **.011** |
|  | Heavy work | 1.221 | 2 | .543 |
|  | Hotter Indoors | .988 | 2 | .610 |
| Dizziness | WBGTmax | 56.586 | 1 | **<.001** |
|  | Cohort | 4.664 | 1 | **.031** |
|  | Maternal Age | 1.797 | 4 | .773 |
|  | Parity | 11.433 | 4 | **.022** |
|  | General Health | .652 | 2 | .722 |
|  | Type of house | 4.423 | 3 | .219 |
|  | Heavy work | 4.842 | 2 | .089 |
|  | Hotter Indoors | 4.446 | 2 | .108 |
| Thirst | WBGTmax | 100.245 | 1 | **<.001** |
|  | Cohort | 1.663 | 1 | .197 |
|  | Maternal Age | 8.149 | 4 | .086 |
|  | Parity | 3.084 | 4 | .544 |
|  | General Health | 1.937 | 2 | .380 |
|  | Type of house | 1.891 | 3 | .595 |
|  | Heavy work | 4.562 | 2 | .102 |
|  | Hotter Indoors | 7.859 | 2 | **.020** |
| Exhaustion | WBGTmax | 99.972 | 1 | **<.001** |
|  | Cohort | .563 | 1 | .453 |
|  | Maternal Age | .990 | 4 | .911 |
|  | Parity | 17.467 | 4 | **.002** |
|  | General Health | 2.762 | 2 | .251 |
|  | Type of house | 16.002 | 3 | .**002** |
|  | Heavy work | .967 | 2 | .617 |
|  | Hotter Indoors | .242 | 2 | .886 |

***Table S11****. Unadjusted GEE model for five heat strain symptoms: skin rash, sleep disturbance, dizziness, thirst and exhaustion: stratified by pregnant and postpartum participants, using both WBGTmax and Tmax as exposure measures. After Bonferroni correction, all effect estimates were significant except for WBGTmax for skin rash, and Tmax for skin rash in postpartum cohort only.*

| Heat Symptom | Cohort | Variable | B | SE | Wald χ² (df) | p |
| --- | --- | --- | --- | --- | --- | --- |
| Skin Rash | pregnant | 14-day averaged max WBGT | .105 | .0431 | 5.932 (1) | .015 |
|  |  | 14-day averaged Tmax | .142 | .0426 | 11.101 (1) | <.001 |
|  | postpartum | 14-day averaged max WBGT | .113 | .0444 | 6.448 (1) | .011 |
|  |  | 14-day averaged Tmax | .051 | .0330 | 2.367 (1) | .124 |
| Sleep Disturbance | pregnant | 14-day averaged max WBGT | .270 | .0408 | 43.594 (1) | <.001 |
|  |  | 14-day averaged Tmax | .206 | .0312 | 43.655 (1) | <.001 |
|  | postpartum | 14-day averaged max WBGT | .234 | .0315 | 55.242 (1) | <.001 |
|  |  | 14-day averaged Tmax | .281 | .0367 | 58.540 (1) | <.001 |
| Dizziness | pregnant | 14-day averaged max WBGT | .236 | .0409 | 33.433 (1) | <.001 |
|  |  | 14-day averaged Tmax | .217 | .0376 | 33.284 (1) | <.001 |
|  | postpartum | 14-day averaged max WBGT | .197 | .0365 | 29.253 (1) | <.001 |
|  |  | 14-day averaged Tmax | .173 | .0330 | 27.381 (1) | <.001 |
| Thirst | pregnant | 14-day averaged max WBGT | .300 | .0394 | 58.042 (1) | <.001 |
|  |  | 14-day averaged Tmax | .300 | .0428 | 48.862 (1) | <.001 |
|  | postpartum | 14-day averaged max WBGT | .218 | .0301 | 52.391 (1) | <.001 |
|  |  | 14-day averaged Tmax | .246 | .0332 | 54.650 (1) | <.001 |
| Exhaustion | pregnant | 14-day averaged max WBGT | .320 | 0.385 | 68.911 (1) | <.001 |
|  |  | 14-day averaged Tmax | .344 | .0408 | 70.938 (1) | <.001 |
|  | postpartum | 14-day averaged max WBGT | .262 | .0351 | 55.493 (1) | <.001 |
|  |  | 14-day averaged Tmax | .290 | .0395 | 53.936 (1) | <.001 |

***Table S12****. Adjusted GEE model for five heat strain symptoms: skin rash, sleep disturbance, dizziness, thirst and exhaustion: stratified by pregnant and postpartum participants, with Tmax as exposure measure. All effect estimates were significant except for Tmax on skin rash in postpartum cohort.*

| **Heat Symptom** | **Cohort** | **B** | **SE** | **Wald χ² (df)** | **p** |
| --- | --- | --- | --- | --- | --- |
| Skin Rash | pregnant | .138 | .0479 | 8.287 (1) | .004 |
|  | postpartum | .051 | .0382 | 1.755 (1) | .185 |
| Sleep Disturbance | pregnant | .211 | .0353 | 35.699 (1) | <.001 |
|  | postpartum | .308 | .0422 | 53.466 (1) | <.001 |
| Dizziness | pregnant | .268 | .0481 | 30.973 (1) | <.001 |
|  | postpartum | .187 | .0365 | 26.252 (1) | <.001 |
| Thirst | pregnant | .317 | .0498 | 40.427 (1) | <.001 |
|  | postpartum | .252 | .0349 | 52.354 (1) | <.001 |
| Exhaustion | pregnant | .383 | .0495 | 59.936 (1) | <.001 |
|  | postpartum | .302 | .0436 | 47.771 (1) | <.001 |

***Figure S1a****. Forest plot indicating the Odds Ratio (OR) and confidence intervals for the adjusted models for pregnant cohort using Tmax and applying Bonferroni correction accounting for multiple testing. All five heat strain symptoms are significant.*

***Figure S1b****. Forest plot indicating the Odds Ratio (OR) and confidence intervals for the adjusted models for postpartum cohort using Tmax. Four of five heat strain symptoms are significant, after using the Bonferroni correction that accounts for multiple testing.*

***Table S13****. Adjusted GEE model for all five heat symptoms: skin rash, sleep disturbance, dizziness, thirst and exhaustion for a pooled pregnant and postpartum cohort. Effect estimates show similar results comparing the model using 14-day averaged Tmax and 14-day averaged max WBGT.*

| Heat Symptom | Variable | B | SE | Wald χ² (df) | p | OR | 95% CI for OR |
| --- | --- | --- | --- | --- | --- | --- | --- |
| Skin Rash | 14-day averaged max WBGT | .110 | .031 | 12.511 (1) | <.001 | 1.116 | 1.050- 1.185 |
|  | 14-day averaged Tmax | .087 | .0261 | 11.020 (1) | <.001 | 1.091 | 1.037- 1.148 |
| Sleep Disturbance | 14-day averaged max WBGT | .250 | .0251 | 99.121 (1) | <.001 | 1.284 | 1.223- 1.349 |
|  | 14-day averaged Tmax | .241 | .0235 | 105.209 (1) | <.001 | 1.273 | 1.215- 1.332 |
| Dizziness | 14-day averaged max WBGT | .214 | 0.027 | 62.516 (1) | <.001 | 1.239 | 1.175- 1.306 |
|  | 14-day averaged Tmax | .191 | .0247 | 59.909 (1) | <.001 | 1.210 | 1.154- 1.271 |
| Thirst | 14-day averaged max WBGT | .254 | .0242 | 110.033 (1) | <.001 | 1.290 | 1.229- 1.351 |
|  | 14-day averaged Tmax | .265 | .0259 | 104.713 (1) | <.001 | 1.303 | 1.240- 1.372 |
| Exhaustion | 14-day averaged max WBGT | .286 | .0259 | 112.49 (1) | <.001 | 1.331 | 1.266-1.401 |
|  | 14-day averaged Tmax | .305 | .0281 | 117.664 (1) | <.001 | 1.357 | 1.284- 1.433 |

***Table S14.*** *Receiver operating characteristics (ROC) analysis identifying Tmax thresholds for heat strain symptoms among pregnant and postpartum participants. AUC: area under the curve. The null hypothesis for ROC analysis is AUC=0.5, indicating no discriminatory ability.*

| **Heat Strain Symptom** | **Cohort** | **AUC** | **95% CI** | **Optimal Tmax cutoff** |
| --- | --- | --- | --- | --- |
| **Skin Rash** | pregnant | 0.635 | 0.543 – 0.726 | 24.88 |
|  | postpartum | 0.520 | 0.433 – 0.606 | 24.50 |
| **Sleep Disturbance** | pregnant | 0.700 | 0.637 – 0.764 | 23.09 |
|  | postpartum | 0.788 | 0.734 – 0.842 | 25.39 |
| **Dizziness** | pregnant | 0.706 | 0.642 – 0.770 | 24.59 |
|  | postpartum | 0.697 | 0.630 – 0.763 | 24.82 |
| **Thirst** | pregnant | 0.770 | 0.706 – 0.833 | 24.29 |
|  | postpartum | 0.764 | 0.705 – 0.823 | 24.61 |
| **Exhaustion** | pregnant | 0.790 | 0.734 – 0.847 | 21.80 |
|  | postpartum | 0.797 | 0.743 – 0.851 | 25.39 |

***Table S15****. USG data, stratified amongst pregnant and postpartum cohort, associated with WBGTmax and Tmax, indicating that WBGTmax was not significantly associated with USG data, while maximum temperature was significantly associated with USG data.*

| Predictor | Cohort | *ρ* | *p* | N |
| --- | --- | --- | --- | --- |
| WBGTmax | pregnant | 0.041 | .508 | 262 |
|  | postpartum | 0.069 | .260 | 266 |
| Tmax | pregnant | 0.124 | .045 | 262 |
|  | postpartum | 0.259 | <.001 | 266 |

***Table S16.*** *Random effect generalised linear mixed method mode assessing sweating sensation. Reference category: dry (1).*

| **Between-participant heterogeneity** | | | | |
| --- | --- | --- | --- | --- |
| Sweat Rating | Estimate | SE | Z | p |
| a little wet | 1.31 | .191 | 6.90 | <.001 |
| wet | 1.64 | .232 | 7.06 | <.001 |
| extremely wet | 3.06 | .473 | 6.47 | <.001 |

***Figure S2****. Number of responses for in-app skin wetness / sweating response.*

***Figure S3a****. Probability thresholds of experiencing heat strain symptom at various probability increases for pregnant cohort, corresponding to specific WBGTmax temperatures. NR stands for not reported, since thresholds lie outside of our observed temperature range of 16 to 28.8°C WBGT.*

***Figure S3b****. Probability thresholds of experiencing heat strain symptom at various probability increases for postpartum cohort, corresponding to specific WBGTmax temperatures. NR stands for not reported, since thresholds lie outside of our observed temperature range of 16 to 28.8°C WBGT.*

***Table S17****. Detailed questionnaire and in-app survey items, with their respective answering options. Questionnaire was administered three times across a six-month period (baseline, follow-up (2 months), endline (6 months)), and in-app survey item could be filled out continuously throughout the intervention period.*

| **Questionnaire and in-app survey items** | | |
| --- | --- | --- |
| **Data source** | **Heat Symptom** | **Answer option** |
| Questionnaire | Over the past two weeks, have you suffered from skin rashes because of the heat? | Yes / No (binary) |
|  | Over the past two weeks, have you had difficulties sleeping because of the heat? | Yes / No (binary) |
|  | Over the past two weeks, have you felt dizzy because of the heat? | Yes / No (binary) |
|  | Over the past two weeks have you felt intensely thirsty because of the heat | Yes / No (binary) |
|  | Over the past two weeks have you ever felt so completely exhausted from the heat that you have been unable to carry on with your normal daily activities? | Yes / No (binary) |
| In-app survey | Please rate your current level of wetness due to sweating | 1: dry, 2: a little wet, 3: wet, 4: extremely wet |
